# Combining time domain modulation optofluidics and high dynamic range imaging for multiplexed, high throughput digital droplet assays

**DOI:** 10.1038/s41378-025-00918-2

**Published:** 2025-05-16

**Authors:** Yasemin Atiyas, Michael J. Siedlik, Stephanie J. Yang, David A. Issadore

**Affiliations:** 1 Department of Bioengineering, University of Pennsylvania, Philadelphia, PA 19104 USA; 2 InfiniFluidics Inc., Philadelphia, PA 19146 USA; 3 Department of Electrical and Systems Engineering, University of Pennsylvania, Philadelphia, PA 19104 USA

**Keywords:** Electrical and electronic engineering, Optical sensors

## Abstract

Digital enzyme-linked immunoassays (dELISA) have been successfully applied to the ultrasensitive quantification of analytes, including nucleic acids, proteins, cells, and extracellular vesicles, achieving robust detection limits in complex clinical specimens such as blood, and demonstrating utility across a broad range of clinical applications. The ultrasensitivity of dELISA comes from partitioning single analytes, captured onto a microbead, into millions of compartments so that they can be counted individually. There is particular interest in using dELISA for multiplexed measurements, but generating and detecting the billions of compartments necessary to perform multiplexed ultrasensitive dELISA remains a challenge. To address this, we have developed a high-throughput, optofluidic platform that performs quantitative fluorescence measurements on five populations of microbeads, each encoded with distinct ratios of two fluorescent dyes, for digital assays. The key innovation of our work is the parallelization of droplet generation and detection, combined with time-domain encoding of the excitation sources into distinct patterns that barcode the emission signal of both dyes within each bead, achieving high throughput (6 × 10^6^ droplets/min) and accurate readout. Additionally, we modulate the exposure settings of the digital camera, capturing images of multiplexed beads and the droplet fluorescent substrate in consecutive frames, a method inspired by high dynamic range (HDR) photography. Our platform accurately classifies five populations of dual-encoded beads (accuracy > 99%) and detects bead-bound streptavidin-horseradish peroxidase molecules in a third fluorescence channel. This work establishes the technological foundation to combine high multiplexing and high throughput for droplet digital assays.

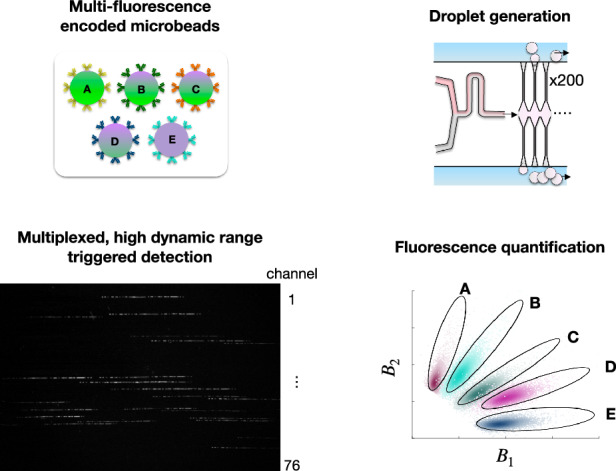

## Introduction

Digital enzyme-linked immunoassays (dELISA) have generated substantial academic and commercial interest for biomarker detection due to their remarkable performance advantages compared to conventional ELISA, including a ~1000-fold improvement in the limit of detection (LOD) and limit of quantitation (LOQ), enhanced robustness across diverse reaction conditions, and absolute quantitation^1,2^. In addition to proteins, dELISA has been extended to the ultrasensitive detection of nucleic acids^3^, mammalian cells^4^, bacteria^5^, viruses^6^, and extracellular vesicles^7,8^. This enhanced performance is achieved by counting individual biological quanta (i.e., proteins, nucleic acids, vesicles, or cells) loaded into many femtoliter-sized compartments, such that there is a ‘digital’ quantity (i.e., 1 or 0) within each compartment. The advantages of dELISA make it well-suited to detect exceedingly sparse concentrations of biomarkers in a wide range of specimens, in areas as far ranging as infectious disease^9^, cardiovascular diseases^10^, cancer^11^, and neurological diseases^12^. The clinical utility of measuring these biomarkers has been validated across a broad range of pre-clinical and patient cohorts^13–21^, and digital assay technology has matured to the point of being successfully commercialized^2^.

There has been an effort to develop microfluidic technology to generate and measure large numbers of microscale droplets to perform dELISA, as LOD, LOQ, and dynamic range (DR) have all been found to improve as the number of compartments increases^7^. The performance of dELISA is a function both of the affinity ligands used, i.e., their affinity to the target and the specificity of that interaction, as well as the size and number of the compartments used. In particular, the LOD and LOQ of dELISA are determined by the number of false positives, i.e., the number of compartments that report a positive signal in the absence of any target molecules. This false positivity is typically dominated by non-specific binding of the labeling affinity ligand to the solid substrate^22^. As dELISA has evolved over the years, there has been progress in reducing this false positivity rate to values as low as ~0.01% false positive droplets, i.e., the fraction of droplets that are positive for a blank sample where there are no target analytes^7,8^. At these low false positive rates, the performance of the assay can be improved by increasing the number of droplets analyzed, up until the point that the uncertainty in the measurements of a blank signal (i.e., the number of positive droplets in the absence of target) ceases to be dominated by Poisson error^7^. For assays with 0.01% false positive rates, performance has been shown to continue to improve for up to ~10 million droplets per assay^7^.

Relatedly, as the biomarker field matures, it faces fundamental limitations in the clinical sensitivity and specificity of single biomarkers, driving growing interest in multiplexed assays. Underlying this issue is the fact that the phenotypic heterogeneity of many diseases and the variability in baseline biomarker expression among healthy individuals often limit clinical sensitivity and specificity. To address this issue, there is a growing appreciation that multiplexed, and increasingly multimodal measurements (i.e., different classes of biomarkers, such as proteins, EVs, nucleic acid), can capture a more comprehensive state of the patient’s disease state than any single measure and mitigate the challenges of subject-to-subject variability^23–25^. There has been much progress in the development of conventional multiplexed protein assays that use flow cytometry as a readout, wherein beads with distinct antibody coatings are color-coded using ratios of multiple fluorescent dyes. Indeed, these approaches have established as much as 500-plexed bead arrays for conventional ELISA applications^26,27^. This paper focuses on the technological challenge that emerges when one attempts to combine high multiplexing with ultra-high sensitivity digital droplet assays. Because each ultrasensitive dELISA assay requires ~10 million droplets, an N-plex assay will require N*10^7^ droplets to be analyzed, which is currently challenging due to the throughput limitations of conventional microfluidic or static partitioning methods^28,29^.

**Fig. 1:**
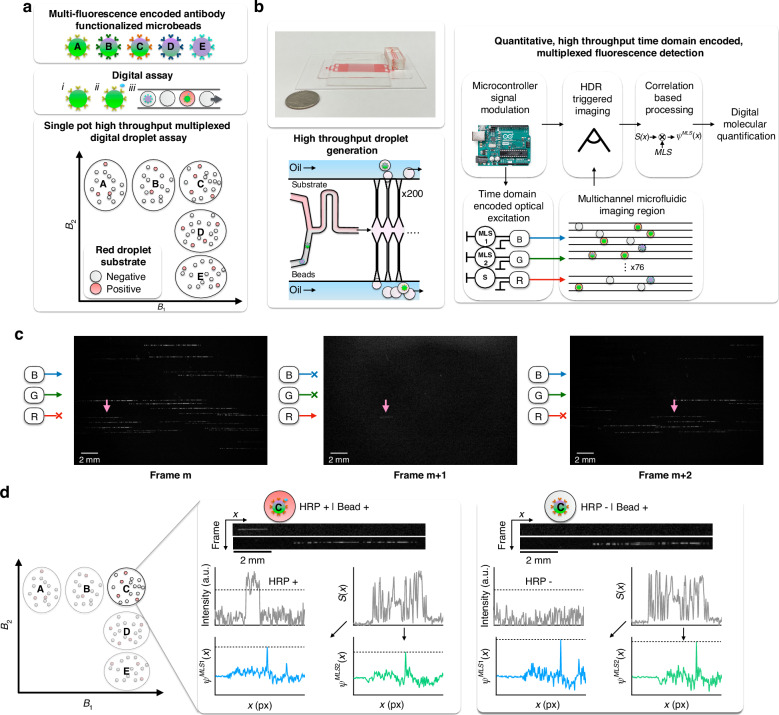
Overview of XµMD. **a** XµMD performs the quantitative fluorescence measurements of 5 populations of dual-encoded beads for digital ELISA applications. **b** The XµMD device, which combines a Millipede geometry for high-throughput droplet generation (bottom) with parallelized droplet detection (right) using HDR imaging, followed by correlation-based analysis to quantify bead fluorescence and detect positive droplets. This approach transfers the complexity of quantifying multiple fluorescence intensities from hardware to software. **c** Overview of HDR imaging, wherein encoded bead and substrate fluorescence signals are interrogated in subsequent frames via modulated exposure, gain, and laser excitation. **d** Summary of image analysis and signal decoding that shows examples of the raw droplet image (top row), the channel intensity line average S(x) (middle row), and correlation results (bottom row) for a droplet that contains a bead and an enzyme (left), and a droplet that contains a bead without an enzyme (right)

In this work, we develop an optofluidic platform for multiplexed dELISA based on the high-throughput, quantitative detection of dual-encoded fluorescent beads for multiplexed digital assays (Fig. 1). We coin this technology multiple**X**ed **micro**droplet **M**egascale **D**etector (XμMD). XµMD overcomes the conventional throughput limitations of microfluidics by parallelizing droplet generation^30^ and detection, and by quantitatively interrogating the fluorescence of each droplet and the bead that it encapsulates in-flow with a time-domain encoded excitation scheme (Fig. 1a, b), building on the framework of an earlier technology from our lab^31^. In our previous work, the fluorescent microbeads and droplets were identified by the presence or absence of a fluorescence signal. Each antibody-functionalized bead population was labeled with a different fluorophore, and thus it was only possible to perform a 2-plex assay, using two fluorescence channels to identify the beads and a third fluorescence readout channel for the droplet fluorescent substrate^7,8,32^. In contrast to our prior work, XµMD makes a quantitative measurement of the fluorescence of each bead instead of a digital one, allowing multiple populations of dual-encoded fluorescent beads that are each encoded with distinct ratios of blue and green dyes to cover the span of a two-dimensional intensity map (Fig. 1a). Our optical system is designed using low-cost, accessible hardware, including a digital camera, a single emission filter, a low-cost three laser diode module, and a microcontroller to coordinate the time-domain modulated light sources and camera (Fig. 1c). The key to our performance is transferring the complexity of measuring multiplexed fluorescence signals from hardware to software by performing correlation analysis, from which we can decode and quantify the individual blue and green fluorescent intensities of each bead (Fig. 1d). In addition to modulating the fluorescent signals in the time domain, XµMD decouples the parameters needed to quantitatively identify the bead with the digital problem of detecting the fluorescence signal of the droplet substrate by modulating the exposure settings of the camera in time, an approach akin to high dynamic range (HDR) photography, where multiple images taken with different exposure settings are combined computationally to synthetically increase the dynamic range of a photograph (Fig. 1c, d). In this work, we demonstrate that our platform can distinguish five populations of dual-encoded multiplexed beads, named Groups A-E, with comparable accuracy to flow cytometry (accuracy > 99%) and can also detect the presence or absence of single streptavidin-horseradish peroxidase (HRP) molecules within the droplets, as a model system for dELISA, at a throughput of 6 × 10^6^ droplets/minute.

## Results

### Optical design for multiplexed fluorescence detection

**Fig. 2:**
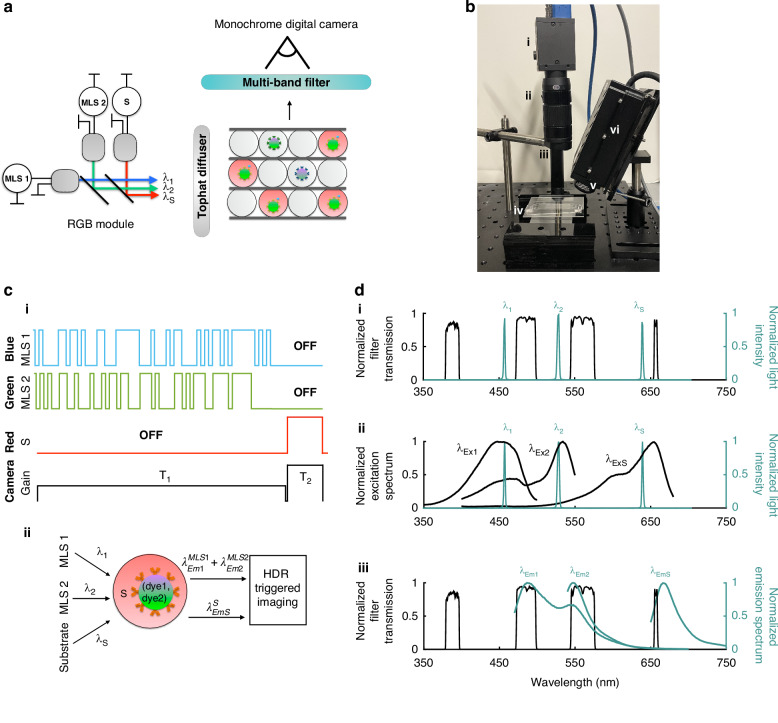
Optical design and signal encoding of XµMD. **a** Schematic of our optical setup, which consists of a laser module that houses a blue, green, and a red laser, a tophat diffuser, a multi-band filter and a monochrome digital camera. **b** Photograph of the setup, which includes the **i** camera, **ii** zoom lens, **iii** bandpass filter, **iv** detection chip, **v** diffuser, and **vi** laser module. **c** Our signal encoding and decoding scheme, wherein the blue and green lasers are modulated by their respective MLS sequences to interrogate the bead, followed by the red laser that interrogates the fluorescent droplet, synchronized with modulated camera exposure and gain settings that separates the interrogation of the bead and substrate into consecutive frames (**i**). As a result, the emission signals of the blue and green dyes are barcoded with their respective MLS patterns and superimposed in one frame, and the red substrate fluorescence is recorded as a digital “on/off” signal in the subsequent frame (**ii**). **d** Overview of our optical design components. **i** The bandpass filter blocks the excitation wavelengths. **ii** The laser wavelengths overlap with the excitation spectra of the three dyes. **iii** The bandpass filter transmits the emission spectra of the three dyes

To achieve high-throughput detection of multiplexed fluorescence signals, XµMD’s optical setup is designed to measure the fluorescence of droplets in-flow through 76 parallel channels, across a total field of view FOV = 350 mm^2^ (Fig. 2). The droplets encapsulate dual fluorescence encoded microbeads, which contain a blue (λ_Ex1_/λ_Em1_ = 448/488 nm) and a green dye (λ_Ex2_/λ_Em2_ = 534/548 nm), and the fluorescent substrate in the droplet contains a far-red dye (λ_ExS_/λ_EmS_ = 654/667 nm) that fluoresces upon reacting with HRP. The three dyes are interrogated using a blue (λ_Blue_ = 457 nm), green (λ_Green_ = 528 nm) and a red (λ_Red_ = 639 nm) laser, housed within a compact module (Techhood) and modulated in time using a microcontroller (Arduino Due). The laser beams exit the module through a single aperture and are expanded using a 20° top-hat diffuser (Thorlabs) to uniformly illuminate the FOV that encompasses the detection region of our microfluidic chip. The detection region consists of 76 parallel soft lithography defined microfluidic channels spaced 110 µm apart, where each channel has a cross-section of 90 × 90 µm^2^ and is imaged along a channel length of 23 mm. The fluorescence emission signals pass through a multi-bandpass filter (λ_cwl_ = 485 ± 10 nm, 559 ± 12.5 nm, 649 ± 6.5 nm) and notch filter in series (λ_cwl_ = 642 ± 13 nm, optical density 6), selected to transmit the dye emission wavelengths and block the excitation wavelengths, before being imaged by a monochrome digital camera (Fig. 2a, b, d).

### Time-domain modulation of the multiplexed dELISA signal

We adapt a time-domain modulation approach, previously developed by our lab^31^, wherein we modulate the excitation sources using maximum length sequences (MLS) and the camera’s exposure settings, and subsequently demodulate the image in software to read out our multiplexed dELISA signal. We use MLS sequences because they are algorithmically defined to have minimum autocorrelation and cross correlation^33^. This time domain modulation approach is designed to allow us to: (1) distinguish the fluorescence emission from two dyes excited with light sources tuned to their respective absorption spectra, each modulated with a different MLS, (2) resolve the distinct fluorescence signals of beads whose images overlap in space due to droplet flow and the finite exposure time of the camera^31^, and (3) decouple the imaging parameters that are optimized to quantitatively measure the fluorescence of the multiplexed beads from those optimized to digitally detect the fluorescent HRP substrate within the droplets. The camera updates exposure parameters in real time to distinct settings for measuring the beads or droplets, in sync with the two light sources used to excite the microbeads (blue λ_Blue_ = 457 nm and green λ_Green_ = 528 nm) and the light source used to excite the droplets (red λ_Red_ = 639 nm) (Fig. 2c). To measure the microbeads, we modulate the blue and green lasers with two distinct 63-bit MLS sequences (MLS1 for blue and MLS2 for green) to encode the beads’ blue and green dye emissions as a superimposed streak a single frame. This is an expansion of our technology from earlier work^7,8,32^, in which a single MLS pattern was sufficient to encode and identify a bead population. We chose to decode these dual-encoded beads as a model system specifically because they contain two dyes that have non-overlapping excitation profiles, so that each dye can be interrogated and barcoded with a different wavelength laser that is modulated with its unique MLS sequence. Most other commercially available multiplexed beads, such as dual-encoded beads manufactured by Luminex, the gold standard, have a single excitation wavelength to excite both dyes^27^, making them unsuitable for our MLS-encoded excitation approach. We choose an exposure time T_1_ = 41.5 ms to image the moving beads so that the length of the imaged streak is ~1/3 the length of the channel, and each bead is imaged at least twice. The exposure time set to measure the fluorescent HRP substrate is T_2_ = 10 ms, during which the red laser is on continuously, chosen to collect sufficient signal from the fluorescent substrate while minimizing the probability of imaging two overlapping streaks from fluorescent droplets. Assuming that maximum 1% of the droplets contain HRP due to Poisson statistics, the positive droplets will be on average 100 droplet diameters, or 8 mm, away from one another. An exposure time of 10 ms results in ~2 mm streaks, minimizing the possibility of imaging two overlapping positive droplets.

### Decoding time-domain encoded multiplexed dELISA signal

**Fig. 3:**
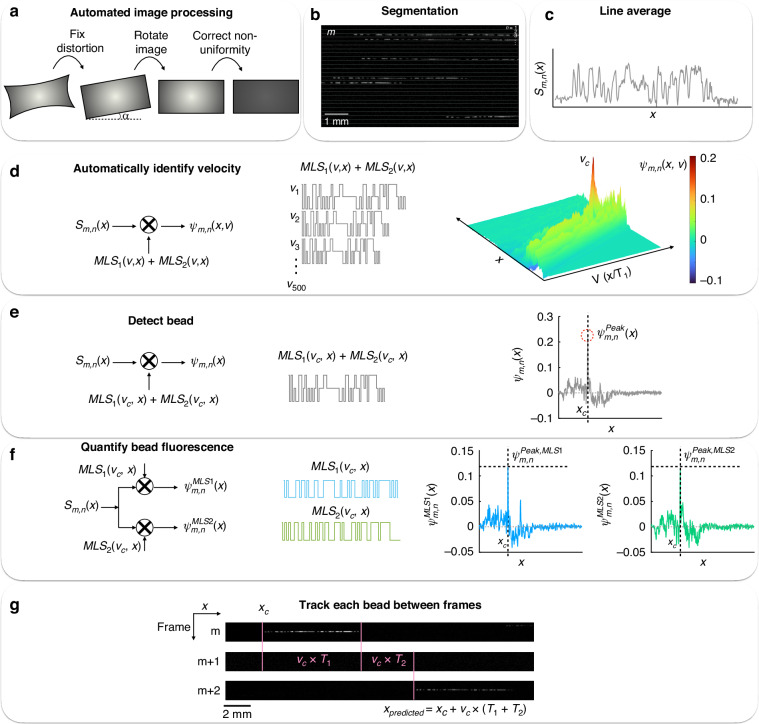
Signal decoding for multiplexed fluorescence measurements. **a** Each frame in a video is corrected for lens distortion, rotated, and corrected for non-uniform illumination via background normalization. **b** Each corrected frame *m* is segmented into *n* = 76 channels, corresponding to the parallel microfluidic channels in the detection region. **c** Each channel is converted into a 1-D vector S_m,n_(x) along channel length x. **d** To identify the velocity of each droplet, the bead signal S_m,n_(x) is correlated with a superimposed mask of MLS 1 and MLS 2 at 500 different velocities. The correct velocity is the one that results in the highest correlation peak. **e** Next, the bead signal is correlated with the superimposed mask at the correct velocity to determine x_c_, the location of the bead in the channel. **f** Finally, knowing the velocity and location of the bead, the bead signal is correlated with individual MLS1 and MLS2 masks to determine the blue and green peak magnitudes at the determined location. **g** We predict the next location of the bead based on its determined location (x_c_), velocity (v_c_), and exposure time. For HDR mode, the exposure time used to image the bead T_1_ and the exposure time used to image the fluorescent droplet T_2_ are both considered when predicting the next location of the bead (x_predicted_), using the formula shown. If a bead is found in that location, the peak magnitudes of the two measurements are averaged for each dye

Our technology transfers the complexity of decoding and quantifying distinct fluorescent signals from hardware to software by performing correlation analysis on the superimposed bead emission signal and extracting individual blue and green dye intensities B_1_ and B_2_ for each bead (Fig. 3). First, we process each frame *m* by correcting for lens aberrations, rotating the image such that the channels are horizontal, and normalizing each frame by a background frame to correct for the spatial non-uniformity of illumination (Fig. 3a, SI Fig. 1). Next, we segment each corrected frame into *n* = 76 channels (Fig. 3b), in which we take a line average along the microfluidic channel width to convert each channel’s fluorescence signal into a 1-D vector, S_m,n_(x)_,_ along the channel length x (Fig. 3c). For a channel that contains a bead, S_m,n_ contains the sum of the fluorescence emissions of the blue and green dye within that bead, λ^MLS1^_Em1_ + λ^MLS2^_Em2_. To detect the presence of a bead and to measure its velocity, necessary to accurately quantify the bead’s fluorescence and the fluorescence of the substrate, we first correlate the channel vector S_m,n_ with a 2-D matrix MLS_1_(v,x)+MLS_2_(v,x), the expected pattern at 500 different velocities, and calculate the correlation matrix Ψ_m,n_(x,v) = S_m,n_(x) ⊗ (MLS_1_(v,x)+MLS_2_(v,x)) (Fig. 3d). To find the velocity v = v_c,_ we identify the highest correlation peak in the 3-D matrix Ψ_m,n_(x,v). After finding v_c_, we analyze the correlation vector at this velocity Ψ^MLS1+MLS2^_m,n_(x,v_c_) to find the location of the peak x = x_c_ (Fig. 3e). Finally, we quantify bead fluorescence by correlating S_m,n_(x) with MLS_1_(v_c_,x) and MLS_2_(v_c_,x) individually and recording the magnitude of the peaks in Ψ^MLS1^_m,n_(x) = S_m,n_(x) ⊗ MLS_1_(v_c_,x) and Ψ^MLS2^_m,n_ (x)= S_m,n_(x) ⊗ MLS_2_(v_c_,x) at each bead location x_c_, using that bead’s measured velocity v_c_ (Fig. 3f). Overall, our processing time is 0.7 s/frame using MATLAB 2021b locally. This computation time can be improved by applying GPU acceleration or by using cloud computing, as previously reported^32^.

To improve the accuracy of our quantification of the blue and green fluorescence of each bead, we apply three post-processing steps to the analysis described above to improve the precision of this measurement: (1) We choose the exposure time and droplet velocity such that each droplet is measured in at least two subsequent frames to allow us to average the intensity of each bead based on two independent measurements. This requires us to be able to track individual droplets from frame to frame, which is made challenging by the presence of multiple droplets in a FOV and the variance in droplet velocity. To accurately track individual beads between frames, we use the measured location and the velocity of the bead in each frame to predict its location in the next frame (Fig. 3g), based on the assumption that there is no significant acceleration of the beads in the timescale of the exposure time of our camera, which we have shown to be true^31^. In our analysis, we only include beads that are detected in subsequent frames and whose intensities are averaged. Compared to including beads detected once, only measuring beads that are averaged lowers the coefficient of variance (CV) of the measured fluorescence intensities of each bead population. The average CV of the measured blue intensity B_1_ for Groups A-E drops from 24% to 20% when we only include beads that are averaged. Similarly, the average CV of the measured green intensity B_2_ drops from 27% to 22% (SI Fig. 2). (2) Unlike flow cytometry, which measures parameters such as forward and side scatter that can be used as gates to discard false signal that is not an actual droplet, our technology can only measure fluorescence. We therefore established a “quality control” metric from our output by calculating the variance of each bead measurement and excluding measurements of beads with individual measurement CVs >20%. This step increases our average classification accuracy from 98.68% to 99.05% (SI Fig. 3). (3) MLS1 and MLS2 are selected to have minimal cross- and auto-correlation. However, for a finite mask length, there is nonzero crosstalk between the two sequences due to the overlapping bits within the 63-bit sequences when both lasers are on. This results in the peaks in Ψ^MLS1^_m,n_(x_c_) and Ψ^MLS2^_m,n_(x_c_) to have a linear correlation coefficient R between 0.7 and 0.9. We apply a correction matrix to account for the proportion of bits when both lasers are on and convert the peaks of Ψ^MLS1^_m,n_(x_c_) and Ψ^MLS2^_m,n_(x_c_) into blue and green intensities B_1_ and B_2_, reducing this crosstalk to comparable levels with flow cytometry measurements (SI Fig. 4).

### Enhancing the dynamic range of measurement

**Fig. 4:**
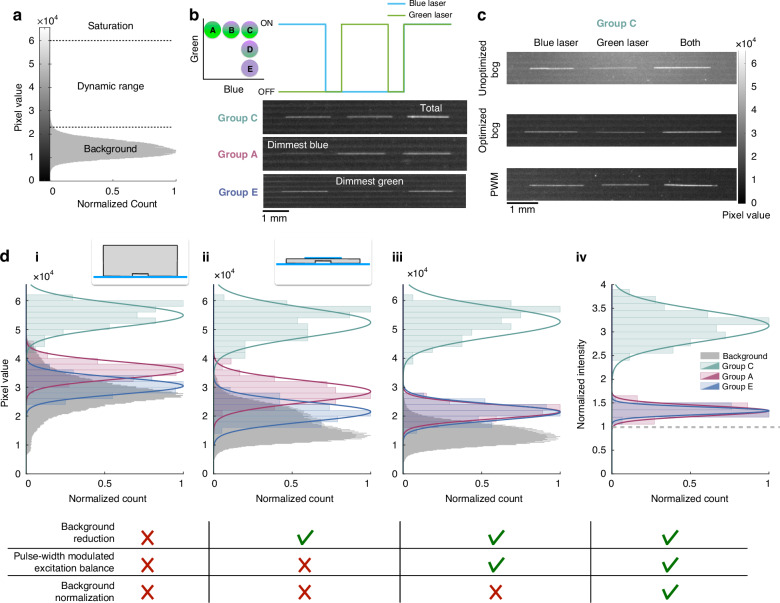
Considerations to enhance the dynamic range of imaging. **a** As we image moving fluorescent beads and droplets using a digital camera, the light that gets collected is converted to a range of pixel values between 0-65,535. Our goal is to resolve the different intensities of the superimposed bead emission signal within this range. **b** Schematic that illustrates our experimental setup to evaluate our dynamic range. To distinguish each bead population’s response to the blue and green lasers, we modulated the excitation sources by first turning the blue laser on, then the green laser on, and finally turning both lasers on. Using this scheme we evaluated Group C’s response to both lasers being on (top of the dynamic range), Group A’s response to the blue laser only, and Group E’s response to the green laser only (bottom of the dynamic range). **c** Example images from Group C demonstrating how the beads’ response to each laser changes by changing the imaging conditions. “bcg”=background,“PWM”=pulse-width modulation. **d** Histograms of average pixel values along the imaged streaks at the top (Group C) and bottom (Group A and E) of the dynamic range, and the pixel values in the absence of beads (background). Transparent bars indicate the raw data, and the solid lines are fits for a Gaussian distribution for N = 80 beads. **i** When the PDMS detection device is unoptimized, the background occupies >40% of the dynamic range, constraining the distance between the top and bottom of the brightest and dimmest dye intensities. Inset shows the unoptimized device, where a 8 mm thick PDMS (gray) is bonded to a glass slide (blue). The primary source of background is laser scattering from the PDMS. **ii** To reduce the background, we decreased the thickness of the detection device to 1 mm, resulting in a decrease in background. Inset shows the optimized device, consisting of a thin PDMS piece sandwiched between two glass slides. **iii** To balance the dimmest blue and green pixel values at the bottom of the dynamic range, we modulated the blue laser using PWM at a duty cycle of 50% to reduce its average power. **iv** Finally, to account for non-uniformity of the excitation, we normalize the bead pixel values by the background signal at the same location in the FOV. As a result, all of the measured bead intensities are above background, where the background is denoted by the gray dashed line

In this work, we found that to accurately quantify multiplexed fluorescence using our MLS approach, the key to performance was to improve the dynamic range of imaging. We image fluorescence signals using a camera that contains 960 × 720 pixels, and each pixel converts the light it collects over an exposure time into a 16-bit number, with values between 0 and 65,535. Within this range, our goal is to detect the superimposed fluorescence intensity from the green and blue dyes corresponding to the identity of the dual encoded microbeads. (Fig. 4a). The top of the dynamic range is occupied by the pixel values that correspond to the MLS sequences when both lasers are on while imaging a bead from group C, which has the highest emission signal for both of the dyes. The bottom of the dynamic range is occupied by the pixel values when only the blue laser is on while imaging group A, and when only the green laser is on while imaging group E, as these two groups contain the lowest emission for the blue and green dyes, respectively. To evaluate the dynamic range and to clearly distinguish the beads’ response to each laser, we imaged the beads in-flow with constant excitation instead of MLS, turning the blue and green laser on sequentially, and then turning both lasers on. We used the same exposure time as we use during our MLS patterning (T = 41 ms) (Fig. 4b). The imaged streaks contained three distinct regions, corresponding to the bead’s response only to the blue laser, only to the green laser, and to both lasers. We quantified the average pixel value within these regions for Groups C, A and E to measure the top and bottom of the dynamic range, and compared their distribution across different conditions (Fig. 4c). We found that the background signal, i.e., the measured light intensity in the absence of beads, mainly caused by laser scattering across the thick (8 mm) PDMS device (Fig. 4di, inset), had an average pixel value of ~27,800 (>40% of the entire dynamic range) prior to any optimization and constrained the distance between the dimmest and brightest signals measured to around half of the available dynamic range (Fig. 4di). To take full advantage of the camera’s dynamic range, it was essential to minimize this background level, which we achieved by fabricating the detection device as a thin (1 mm) layer of PDMS sandwiched between two glass slides (Fig. 4dii, inset, SI Fig. 5). The purpose of bonding a second glass slide on top of the chip was to prevent the channels from delaminating due to the thinness of the PDMS at high flow rates. With the background reduced by 38%, the distance between the brightest and dimmest signals increased; however, the average dimmest blue signal was greater than the average dimmest green signal (28,500 for blue versus 21,500 for green) due to the imbalance between the blue and green laser powers (Fig. 4dii). To correct this imbalance and allocate the dynamic range equally to the blue and green dyes, we modulated the blue laser with pulse-width modulation (PWM) at 9.6 kHz, 6x the frequency of MLS modulation, with a duty cycle of 50% to decrease its average power. With PWM, the background remained low and the dimmest blue and green intensities were both at ~21,500 (Fig. 4diii). Finally, to correct for the spatial non-uniformity of excitation, which contributes to variation in both signal and the background, we normalized each bead signal by its corresponding background signal at the same location in the FOV, resulting in all of the measured signal being above 1, where 1 denotes the background (Fig. 4iv). Overall, the fold-change difference between the average brightest and dimmest signals improved from 1.7 to 2.3 as a result of these subsequent optimization steps.

### Accuracy of classification and comparison to flow cytometry

**Fig. 5:**
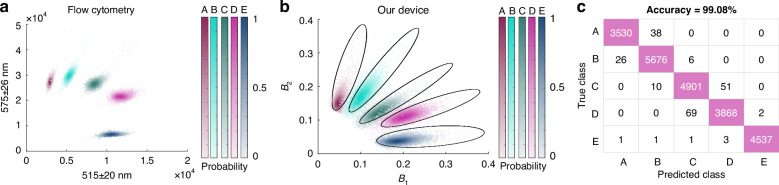
Accuracy of classifying 5 populations of dual encoded beads using XµMD. **a** Flow cytometry measurements of the dual-encoded fluorescent beads, achieving an accuracy of >99.8%. **b** The fluorescence measurements of the same populations of beads using XµMD. Black solid shapes indicate the gates drawn to classify each group. **c** The truth table corresponding to the gates drawn in (**b**), achieving a classification accuracy of 99.08%

We evaluated the performance of our system by quantifying the accuracy of distinguishing five distinct populations of the commercially available fluorescence dye-encoded beads described above in a head-to-head comparison with flow cytometry. We measured each group individually to keep the true group identity known. Using flow cytometry, after gating for singlets using forward and side scatter (Fig. 5a), we manually drew geometrically simple gates to avoid overfitting around each bead population and calculated a classification accuracy for each bead population of >99.8%. Using our platform, following image analysis, peak detection and quality control steps, the beads were distributed along the blue and green intensity space B_1_ and B_2_ with CVs ranging from 17 to 28% (Fig. 5b). Despite this higher variance among individual groups, the accuracy of classification was 99.08%, approaching that of flow cytometry (Fig. 5c), at a throughput >10x greater than flow cytometry. In this work, we used commercially available multiplexed beads to characterize and benchmark our work. Using these beads, our technology can resolve three groups of fluorescent microspheres across both fluorescence channels used (B_1_ and B_2_) within our imager’s dynamic range (Fig. 4). Based on this result, we can separately resolve 3 × 3 = 9 bead sub-populations of these multiplexed beads across a 2-D intensity map using this implementation of our technology. In addition, we note that while this work aimed to quantify the fluorescence of 11 µm, commercially available multiplexed beads, our system is compatible with detecting beads of smaller sizes (<3 µm) that are typically used in digital assays (SI Fig. 6).

### Detection of streptavidin-HRP positive beads using HDR mode

**Fig. 6:**
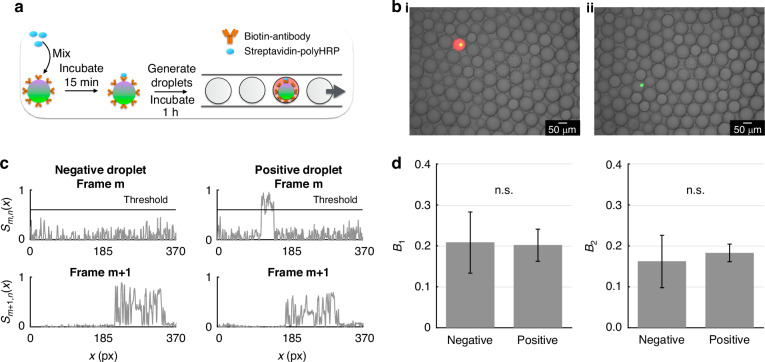
Digital HRP assay. **a** Streptavidin-polyHRP molecules were incubated with Group C beads covalently coupled to biotinylated antibody, washed, and mixed with fluorescent substrate in the Millipede device. After incubating for 1 h, the droplets were reinjected into the detection device for HDR imaging in-flow. **b** Micrographs of **i** a bead that has captured an HRP, resulting in a fluorescent droplet due to the HRP-substrate reaction, and **ii** a bead that has not captured an HRP. **c** 1-D channel vectors corresponding to droplets imaged in consecutive frames *m* (top) and *m* + *1* (bottom). In frame *m*, we detect the digital absence (left) or presence (right) of fluorescent substrate based on a threshold. In frame *m* + *1*, we detect the bead that corresponds to the detected HRP signal in frame *m*. **d** The presence or absence of HRP does not significantly affect the measured fluorescence intensity of the blue (left) or green (right) dye (*N* = 109 negatives, *N* = 5 positives, p >> 0.05)

To evaluate our system’s ability to read out dELISA, as a model system, we detected single streptadivin-HRP molecules bound to beads. To test our platform, we coated bead group C with biotinylated Goat Anti-Rabbit IgG secondary antibody and incubated 500,000 beads with 1:500,000 diluted streptavidin-HRP in 1% bovine serum albumin (BSA) in SuperBlock™ (PBS) Blocking Buffer (Thermo Fisher) to allow for the digital binding of HRP molecules to the bead, allowed the reaction to take place for 15 min at room temperature, washed three times via centrifugation, and resuspended in blocking buffer. Then, we mixed the bead-HRP suspension with the infrared substrate, and partitioned the mixture into droplets using the millipede device (Fig. 6a). We allowed the fluorescence amplification to continue for one hour to allow the fluorescent product to saturate inside the droplets (Fig. 6b). We validated via imaging that the fluorescent substrate remains stable in the droplet for 1 h, without diffusing into neighboring droplets (SI Fig. 7). Next, we re-injected the droplets into the detection device at a rate of 6 × 10^6^ droplets/minute, operating our camera in HDR mode to capture the moving beads and fluorescent droplets in consecutive frames. As a proof-of-concept, we showed that we can distinguish between droplets that contain a bead but no HRP and droplets that contain both (Fig. 6c), and that the location of the MLS streak in the “bead” frame directly follows the end of the substrate streak in the previous frame. In addition, the length of the fluorescent substrate streak is ~1/4 of the MLS streak, matching the 1/4 exposure time used to image the fluorescent droplet versus the beads. To determine whether a bead is positive for substrate fluorescence, we use each bead’s location and velocity to determine the expected location and length of the substrate streak in the preceding and succeeding frame. We quantify the fluorescence intensity by summing the pixel values at the expected location for the signal that comes from the substrate and compare that sum to a predetermined threshold, which we define relative to the background signal in the images as 4 standard deviations above the average background measurement (SI Fig. 8). Finally, we compared the measured B_1_ and B_2_ dye fluorescence of the beads that have captured HRP to beads that have not, and found that the presence of the substrate signal does not impact the measured dye fluorescence intensity in either color (Fig. 6d).

## Discussion

In this work, we address a key challenge in multiplexed digital assays, the need to scale the number of compartments with the number of targets to maintain sensitivity while mitigating Poisson noise. We address this need by parallelizing both droplet generation and detection, and by incorporating large field-of-view optics, time-domain encoding, and HDR imaging to simultaneously detect green and blue dyes in dual-encoded multiplexed beads, achieving a droplet processing rate of 6 million droplets per minute. As a proof-of-concept, we successfully distinguished five populations of dual-encoded beads with >99% accuracy.

This work builds on a growing body of technologies that have aimed to increase the multiplexing capabilities of dELISA. The gold standard technology for dELISA, the Simoa technology by Quanterix^34^, can perform multiplexed protein measurements via encoded fluorescent bead detection over a static array, but measuring beyond 10 targets compromises assay sensitivity due to Poisson counting error, since the finite number of microwells (~200,000) does not scale with the number of multiplexed targets^22,28,29^. Emerging technologies have aimed to address this need for increased compartmentalization using continuous flow systems instead of static arrays. One approach has been to make digital assays compatible with flow cytometry-based readout by replacing conventional enzymatic amplification methods, where an enzyme such as HRP converts a substrate from a non-fluorescent state to a fluorescent one, with either rolling circle amplification (RCA)^35,36^ or tyramide signal amplification (TSA)^37,38^, generating an amplified fluorescent product directly on the target and eliminating the requirement for compartmentalization in microarrays. While these approaches have demonstrated utility, the need for flow cytometry-based readout confines their use to research settings and limits performance because of the throughput <10^4^/s of serially inspecting beads, which would take several days of continual processing for an ultrasensitive, >5 plex assay. Droplet microfluidic systems have the capacity to generate a large number of partitions, having demonstrated as many as 10^8^ droplets/s^39^, and to detect droplets at high throughput, having demonstrated as many as 10^6^ droplets/s, a 100x improvement versus flow cytometry^31^. Moreover, droplet microfluidic based assays have demonstrated compatibility with digital assays, achieving miniaturization for point-of-care diagnostics^40^, and have been used for the digital detection of proteins^32,41,42^, nucleic acids^43–45^, and extracellular vesicles^7,8,46,47^. Notably, recently a 5-plexed digital cytokine assay, wherein droplet dELISA is combined with microscopy-based detection of dual-encoded fluorescent beads, was reported^48^. Moreover, HDR imaging has been used to image static compartments to improve the performance and accessibility of dELISA^49^. Despite their well-established promise, however, conventional droplet generation and detection techniques have not been able to achieve the throughputs or multiplexing abilities to match those necessary for ultrasensitive, multiplexed dELISA.

Building on the foundational developments described here, we note that there are multiple opportunities to further develop our technology. For example, the number of multiplexed targets can be expanded by incorporating additional dyes, with distinct absorption spectra, and excitation sources that align with these spectra, extending the two-dimensional multiplexing scheme demonstrated here into additional dimensions. In this work, the dynamic range of the intensity of fluorescence emissions that we can resolve is limited by the dynamic range of the digital camera used. Future work can address this limitation by continuing to build on the HDR approach introduced in this study, by measuring each bead multiple times in subsequent frames using variable excitation intensities, exposure times, and imaging gain. In the current approach, the number of distinct dyes that can be resolved is limited by the spectral overlap of the excitation absorption of available fluorescent dyes. To address this challenge, prior work has shown that the Förster resonance energy transfers between dyes can be accounted for to enhance fluorescence encoded multiplexing, achieving 580-plex barcoding^50^. Additionally, there have been published developments in the generation of highly monodisperse bead populations using parallelized microfluidics^39^ and layer-by-layer assembly^51^ to minimize the size and intensity variance between beads, which can improve multiplexing. Additionally, multiplexing can also be improved by differentiating beads based on their emission spectra as well, and not solely their excitation spectra as we did in this work. To resolve beads with distinct emission spectra, such as quantum dots^52^ or Luminex beads^27^, we can incorporate more than one camera, each equipped with a unique bandpass filter, each designed to measure the emission from a particular dye. Finally, instead of fluorescence encoded microbeads, we can alternatively use the emerging technology of semiconducting polymer dots (Pdots) with high quantum yields and narrow absorption and emission spectra to fluorescently barcode targets of interest^53^. Our throughput can also be increased further by increasing the number of nozzles in the Millipede device, or by using smaller diameter beads that decrease the minimum step height requirement of the Millipede, thereby decreasing the droplet size^30^ and achieving a greater number of droplets per volume compared to our current device.

This work focuses on the technological development to perform multiplexed, high throughput fluorescence measurements for digital assays. To apply this technology to measure multiplexed analytes in clinical samples, significant assay development work must be completed to develop an optimized single-pot, multiplexed bead-based digital assay, to overcome typical issues such as antibody crosstalk^24,54^. Our group and others have successfully applied digital droplet microfluidics to clinical samples, and our MLS-modulated detection scheme has been applied to point-of-care cell phone-based protein detection^32^, in addition to measuring protein and EVs in clinical samples^7,8,32^. Based on these developments, this multiplexed fluorescence detection technology can be practically applied to the measurement of clinical samples in future work.

Our technology opens up a wide range of applications for digital assays. As new molecular detection technologies emerge, combining digital counting with the quantitative analysis of protein or nucleic acid levels in single extracellular vesicles^55,56^, or cells^57^, our platform provides a scalable solution to significantly increase assay throughput. Importantly, our technology is not limited to droplet digital assays and can also be adapted to scale up droplet-free molecular fluorescence assays (i.e., RCA or TSA) that currently rely on conventional flow cytometry as a readout. This increased capacity offers a key capability to characterize biological heterogeneity^58^ and unravel the unique molecular profiles of individual biological quanta, such as cells and vesicles. Moreover, with the growing appreciation of the capability of machine learning algorithms to reduce multidimensional molecular data into useful classifications, our platform’s multiplexing capabilities hold significant potential. By facilitating the analysis of complex molecular signatures, our technology can contribute to the discovery of intricate molecular mechanisms, leading to more comprehensive measurements of cell biology, disease pathology, and personalized, precision medicine^59^.

## Materials and methods

### Assay reagents

The carboxyl-coated multiplexed beads were purchased from PolyAn (105-12-011, Berlin, Germany). The Streptavidin Poly-HRP80 Conjugate was purchased from Biosynth (65R-S105PHRP, Compton, United Kingdom) and the far red substrate was purchased from AAT Bioquest (Amplite IR 11009, Pleasanton, CA, USA). The biotinylated Goat Anti-Rabbit IgG secondary antibody was purchased from Abcam (ab207995, Waltham, MA, USA). The blocking buffer components, SuperBlock™ (PBS) Blocking Buffer and SuperBlock T20 (PBS) Blocking Buffer were purchased from Thermo Fisher (37515 and 37516), and the BSA was purchased from Millipore Sigma (A7030). All reagents were stored according to their manufacturer’s recommendations.

### Antibody conjugation of multiplexed beads

The carboxylated beads were conjugated with biotinylated secondary antibody using the PolyLink Protein Coupling Kit (24350-1, PolySciences, Warrington, PA, USA) according to the manufacturer’s protocol. Briefly, 3 mg microparticles (~3.6 × 10^6^ beads) were coated with 25 µg antibody following EDAC activation. All wash steps were performed via centrifugation at 15,000 × *g* for 10 min, and the antibody-coated beads were stored in the kit’s storage buffer in 4 °C at a concentration of 9000 beads/µL.

### Optical setup

XµMD’s detection chip is housed in an acrylic holder to position the chip’s detection region under the monochrome digital camera (GS3-U3-28S5M-C, FLIR, Wilsonville, OR, USA). The chip is illuminated with three excitation sources, a blue (λ_Blue_ = 457 nm), green (λ_Green_ = 528 nm) and a red (λ_Red_ = 639 nm) laser that are housed within an RGB module (Techhood) (Fig. 2a, b). The surface under the detection chip is covered with light-absorbing black tape (Acktar Metal Velvet™, purchased from Edmund Optics #12-695) to limit laser light reflection. The laser beams are collimated and exit the module through a single aperture before being expanded by a 20° tophat diffuser (ED1-C20-MD, Thorlabs, Newton, NJ, USA) to a ~ 25 mm diameter circle to illuminate the 350 mm^2^ FOV. The emitted fluorescence signals pass through a bandpass filter (λ_cwl_ = 485 ± 10 nm, 559 ± 12.5 nm, 649 ± 6.5 nm, FF01-387/485/559/649, Semrock, Rochester, NY, USA) and a OD6 notch filter (λ_cwl_ = 642 ± 13 nm, NF03-642E, Semrock) and reach the c-mount macro zoom lens (MLM3X-MP, Computar, USA). The camera’s exposure settings and the excitation sources are modulated using a microcontroller (Arduino Due) to modulate the excitation sources with their unique MLS patterns, and to sync the start of the MLS with the start of exposure (Fig. 2c). The images of the moving droplets are taken using the FlyCap2 software (2.11.3.121; Point Grey Research) in HDR mode, and are saved as a series of images and stored locally for subsequent analysis.

### Multiplexed bead and fluorescent substrate spectra measurements

The excitation and emission spectra of the multiplexed beads and infrared substrate were measured using the FluoroMax-3 Spectrofluorometer with FluorEssence Software (HORIBA Jobin Yvon) (Fig. 2d). For the blue dye, the excitation sweep was performed between 350 and 500 nm, recording the emission at 510 nm. The emission sweep was performed between 470 and 700 nm with the excitation set at 457 nm to match the wavelength of our blue laser. For the green dye, the excitation sweep was performed between 400 and 570 nm, recording the emission at 580 nm. The emission sweep was performed between 540 and 700 nm, with the excitation set at 528 nm to match the wavelength of our green laser. For the infrared substrate, the excitation sweep was performed between 400 and 680 nm, recording the emission at 690 nm. The emission sweep was performed between 650 and 750 nm, with the excitation set at 639 nm to match the wavelength of the red laser. All measurements were performed in duplicate, with a 1 nm increment and 5 nm bandpass slit. The beads and substrate were diluted in PBS, and for each condition, dye-free PBS was measured in duplicate as the background. For each measurement, the average background spectrum was subtracted from the average dye spectrum. The transmission specs of the bandpass and notch filters were retrieved from the manufacturer’s datasheet.

### Fabrication of the millipede droplet generator and detection chip

XµMD is composed of two microfluidic components to achieve high-throughput, multiplexed droplet detection: a 200-nozzle Millipede device^30^ and a parallelized detection chip that consists of 76 parallel channels, where each channel has a cross-section of 90 × 90 µm^2^ (Fig. 1b). All microfluidic chips were microfabricated using standard soft lithography in the Singh Center for Nanotechnology at the University of Pennsylvania. Briefly, SU8 negative photoresist (Kayaku, Westborough, MA, USA) was spin-coated on a silicon wafer, with the spin rate determining the height of the coated resist. The coated wafer was soft baked, exposed to UV light to pattern the SU8, baked post-exposure, and developed. The patterned silicon wafer was silanized (Trichloro(1*H*,1*H*,2*H*,2*H*-perfluorooctyl) silane, Millipore Sigma) in vacuum for 1 h. Next, PDMS was well-mixed at a ratio of 8:1 elastomer to curing agent (Ellsworth, Germantown, WI, USA), poured over the wafer, desiccated for >1 h in vacuum, and baked overnight in a 65 °C oven. The next day, the cured PDMS was cut out of the wafer, and the inlets and outlets were punched using a 1.5 mm biopsy punch (Integra Miltex). The PDMS piece and a glass substrate (26005, Corning) were treated with oxygen plasma (Anatech SCE-106 Barrel Asher) at 30 W for 15 s, bonded, and baked at 65 °C for 1 h. Prior to droplet generation, the devices were treated with 2% silane in HFE-7500 Novec ™ Engineered fluid (3 M, Saint Paul, MN, USA) for 10 min, and flushed with HFE-7500.

### Assembly of the 1 mm thick detection device

To assemble the modified detection device that is thinner than conventional microfluidic chips, ~3 g of 8:1 PDMS was poured over the silicon wafer, distributed along the surface of the wafer so that it is ~1 mm thick, desiccated, and baked, ensuring that the wafer was leveled. Next, two PDMS pieces, roughly 10 × 10 × 5 mm^3^ (lxwxh) each, were cut to be bonded as posts at the inlet and outlet of the chip. While the PDMS was still on the wafer, i.e., covering the features, the back side of the detection region was taped with Kapton tape (KPT-1, Torrance, CA) to protect that area from oxygen plasma. Next, the wafer and the two PDMS pieces were treated with oxygen plasma, the pieces were aligned with the back side of the inlet and outlet features of the wafer, and bonded. The Kapton tape was removed, and the wafer was baked in a 65 °C oven for >1 h. Next, the thin PDMS device with the bonded inlet and outlet posts was cut out, cleaned with tape, and punched. Next, the device and two glass substrates were treated with oxygen plasma. First, the side of the PDMS with the features was bonded to a glass substrate. Next, the second glass substrate was bonded to the back side of the detection region (the region previously protected by Kapton tape), sandwiching the detection region between two glass slides (SI Fig. 5). Overall, the sandwiched PDMS assembly method yields a thin detection device that can withstand high (>300 mL/h) flow rates. Additionally, the Kapton tape protects the back of the detection region during the first plasma treatment, and enables the sandwiched device to be fabricated within a day without compromising the strength of the covalent bond between the PDMS and glass. The bonded device was baked at 65 °C for 1 h and silanized.

### Millipede geometry and droplet generation

The Millipede consists of a droplet generator layer and a taller continuous phase layer to collect the droplets, and was fabricated using 2-layer photolithography: the droplet generation layer consists of 200 nozzles and has a height *h* = 17 µm, selected to be tall enough to encapsulate 11 µm diameter beads, and the continuous phase layer has a height *h* = 160 µm. Operating 200 nozzles in parallel achieves a maximum throughput of 100,000 droplets per second (SI Fig. 9). The Millipede geometry is flow rate invariant, generating ~80 µm droplets across aqueous phase flow rates Φ_aq_ = 40–100 mL/h and continuous phase flow rates Φ_oil_ = 200–400 mL/h, with CVs <8% (SI Fig. 9). For all experiments, QX200™ Droplet Generation Oil for EvaGreen (#1864006, Biorad, Hercules, CA, USA) was used as the continuous phase.

### Multiplexed bead measurements

To evaluate our system’s ability to accurately distinguish the fluorescence of five populations of dual-encoded beads, we measured them serially using XµMD. 60,000 beads per group were diluted in 400 µL blocking buffer (1% BSA in SuperBlock T20 (PBS) Blocking Buffer) and loaded into a disposable 5 mL syringe (Henke-Ject). To model assay conditions, a second syringe containing PBS was loaded as the second aqueous inlet instead of fluorescent substrate. The aqueous syringes and the oil syringe were connected to syringe pumps (Harvard Apparatus Pump 11 Elite) and to the inlets of the Millipede using disposable tubing (AAD04103 Tygon). The outlet of the Millipede was connected to the inlet of the detection chip, positioned under the camera. The oil was injected at a volumetric flow rate Φ_oil_ = 300 mL/h and the aqueous solutions were injected at a flow rate Φ_aq_ = 40 mL/h each, generating droplets with an average diameter of 80.9 µm with a CV of 5.9% (SI Fig. 9). The moving droplets were interrogated using our MLS-encoded excitation sources and imaged with an exposure time of 41.5 ms. The collected images were analyzed using our image analysis software described above, using MATLAB 2021b (MathWorks) (Fig. 3). The output of our analysis code was analyzed on FlowJo 10.10.0 (Becton Dickinson & Company), along with our flow cytometry measurements.

## Supplementary information

Supplementary Information - Revision Marked Up
