# Supplementary material for: Combining time domain modulation optofluidics and high dynamic range imaging for multiplexed, high throughput digital droplet assays
Source: Microsyst Nanoeng. 2025 May 16;11:93. doi: 10.1038/s41378-025-00918-2 (PMC12084528; doi:10.1038/s41378-025-00918-2)
Supplement: Supplementary file 1 — Supplementary Information - Revision Marked Up [file 41378_2025_918_MOESM1_ESM.docx]

**Combining time domain modulation optofluidics and high dynamic range imaging for multiplexed, high throughput digital droplet assays**

Yasemin Atiyas^1^, Michael J. Siedlik^2^, Stephanie J. Yang^1^, David A. Issadore^1,3*^

Supplementary Information

Microsystems & Nanoengineering

**
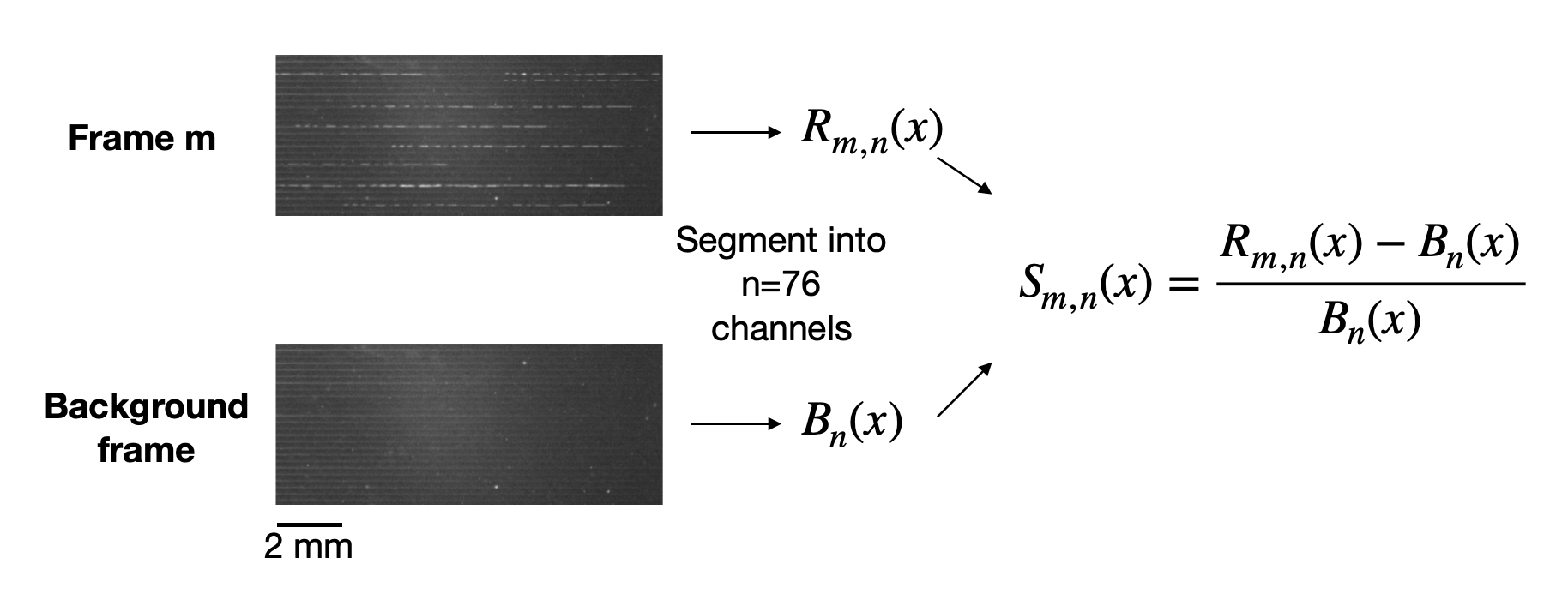
**

**SI Figure 1.** Background normalization. Each 1-D raw channel vector R_m,n_(x) is subtracted and divided by the 1-D background profile corresponding to that channel, B_n_(x). A background frame is selected as a frame that does not contain any beads.


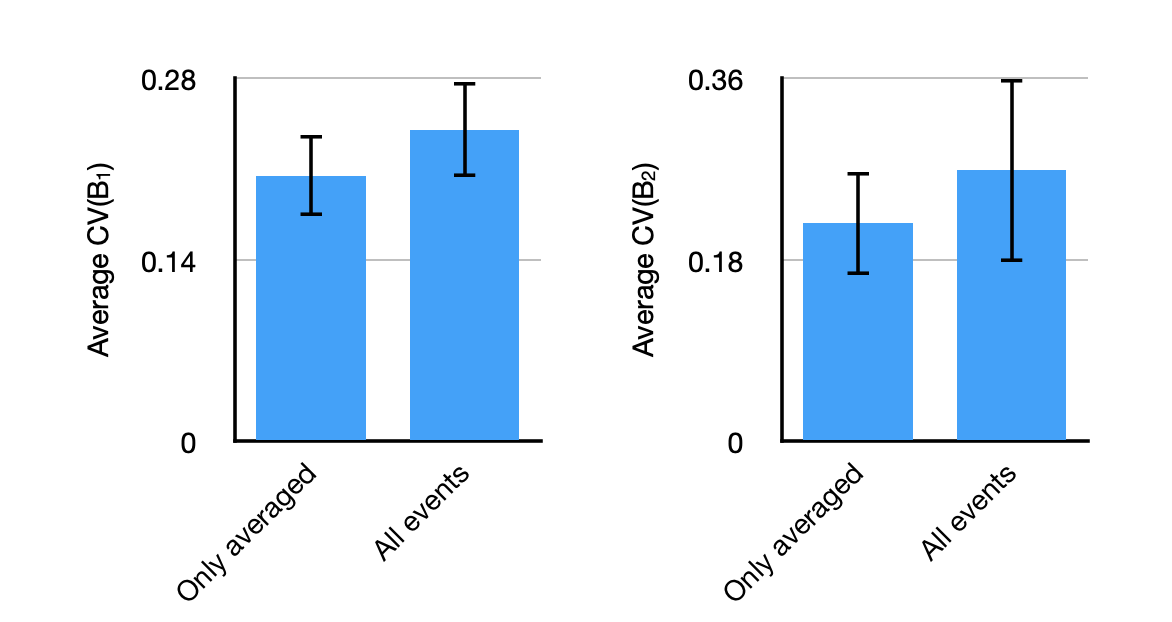


**SI Figure 2.** Peak Averaging. Our peak detection software only measures beads that are detected more than once in subsequent frames and averaged. Compared to measuring all events, *i.e.* including peaks detected once, our approach lowers the average population CV from 24% to 20% for measuring B_1_ and from 27% to 22% for measuring B_2_ across bead populations A-E (Two-tailed paired T-test for Groups A-E, p < 0.05. Error bars represent standard deviation).


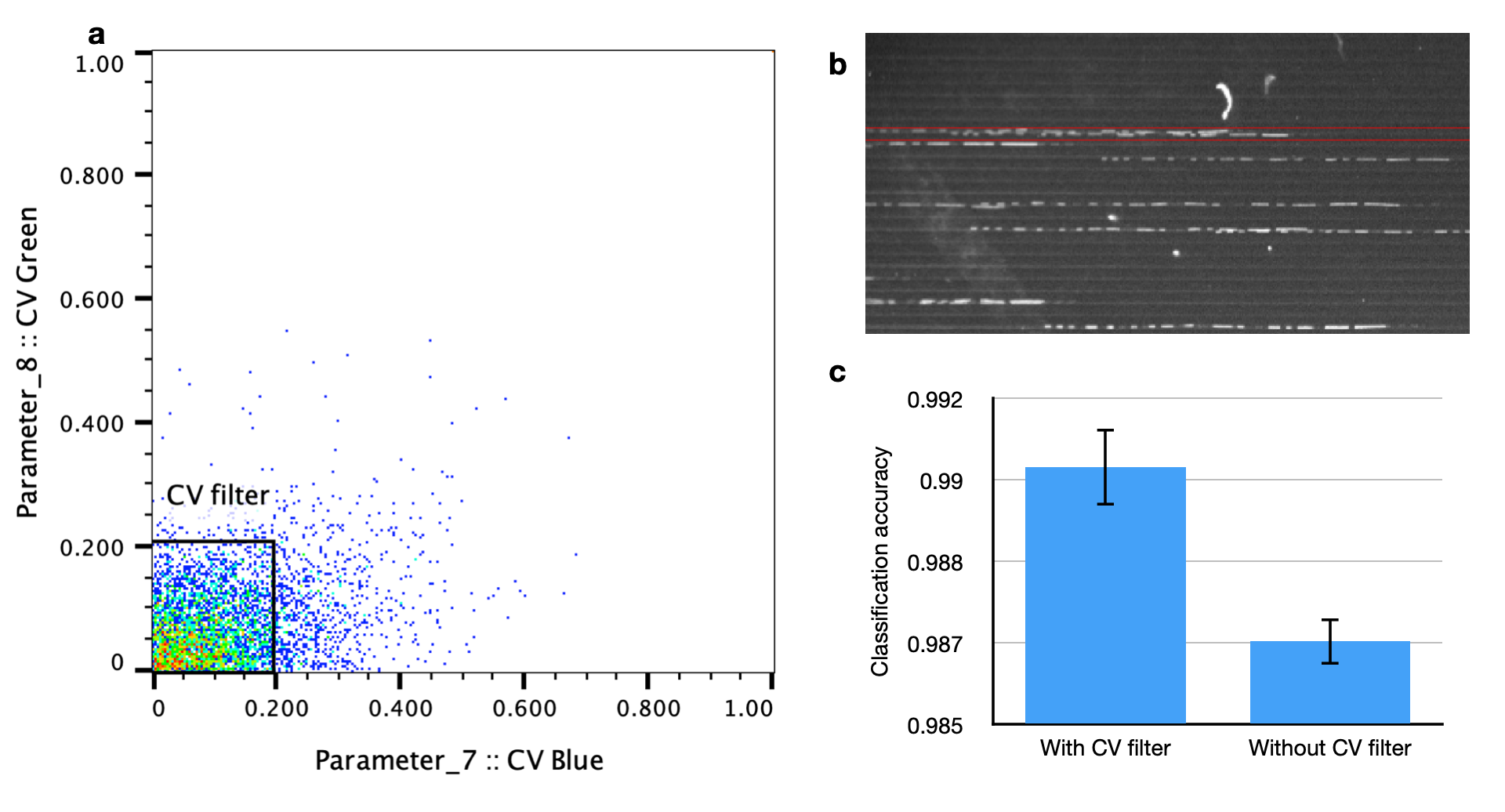


**SI Figure 3.** CV filtering. **a)** CV filtering on FlowJo for quality control enables us to disregard individual bead measurements with CVs > 20%. **b)** CV filtering helps eliminate the measurements of beads within droplets that have non-uniform velocities, as shown as the bead labeled between two red lines. **c)** This approach improves the accuracy of classification. Bar plots show the average classification accuracy when our bead intensity measurements are randomly subsampled into three independent groups, each with N=1600 beads per population, and gated with and without CV filtering (Two-tailed paired T-test, p<0.05. Error bars represent standard deviation).


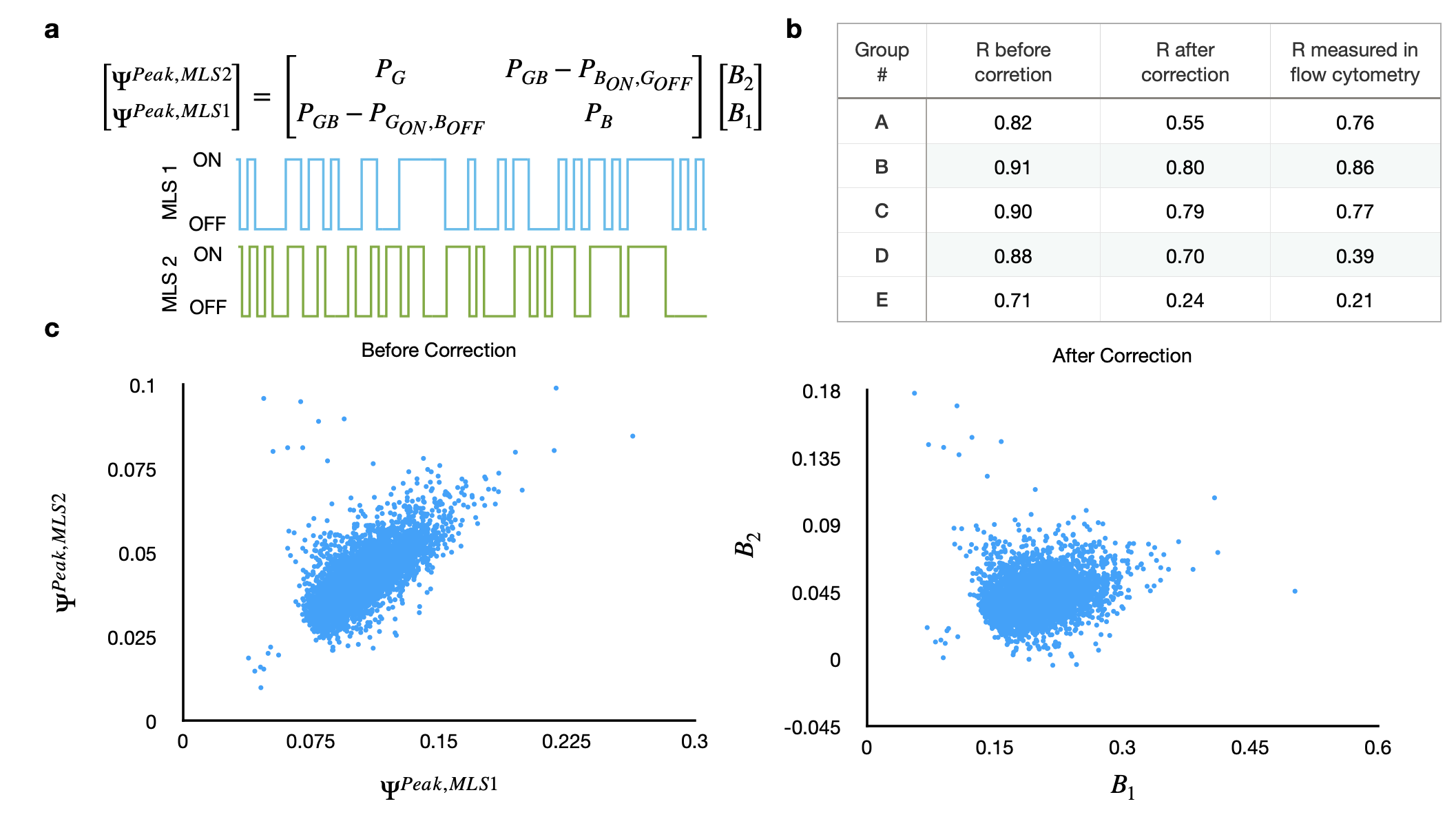


**SI Figure 4.** Correction for MLS crosstalk. **a)** We apply a correction matrix to convert the correlation peak magnitudes Ψ^Peak,MLS1^(x_c_) and Ψ^Peak,MLS2^(x_c_) into blue and green intensities B_1_ and B_2_ for each bead. The correction matrix accounts for the bits that are overlapping within the two 63-bit MLS patterns. P_G_ = proportion of “on” bits in the green MLS mask (MLS 2), P_B_ = proportion of “on” bits in the blue MLS mask (MLS 1), P_GB_ = proportion of overlapping bits in MLS1 and MLS 2, *i.e.* the bits are “on” at the same time, P_Gon,Boff_ = proportion of bits that are “on” in MLS 2 while “off” in MLS 1, P_Bon,Goff_ = proportion of bits that are “on” in MLS 1 while “off” in MLS 2. **b)** Applying this correction decreases the linear correlation coefficient R between the two dye intensity measurements, and approaches the R values measured by flow cytometry. **c)** Scatterplot of the measured fluorescence intensities of Group E before and after correcting for MLS crosstalk, showing that the linearity between the two dye intensities is diminished after applying the correction.


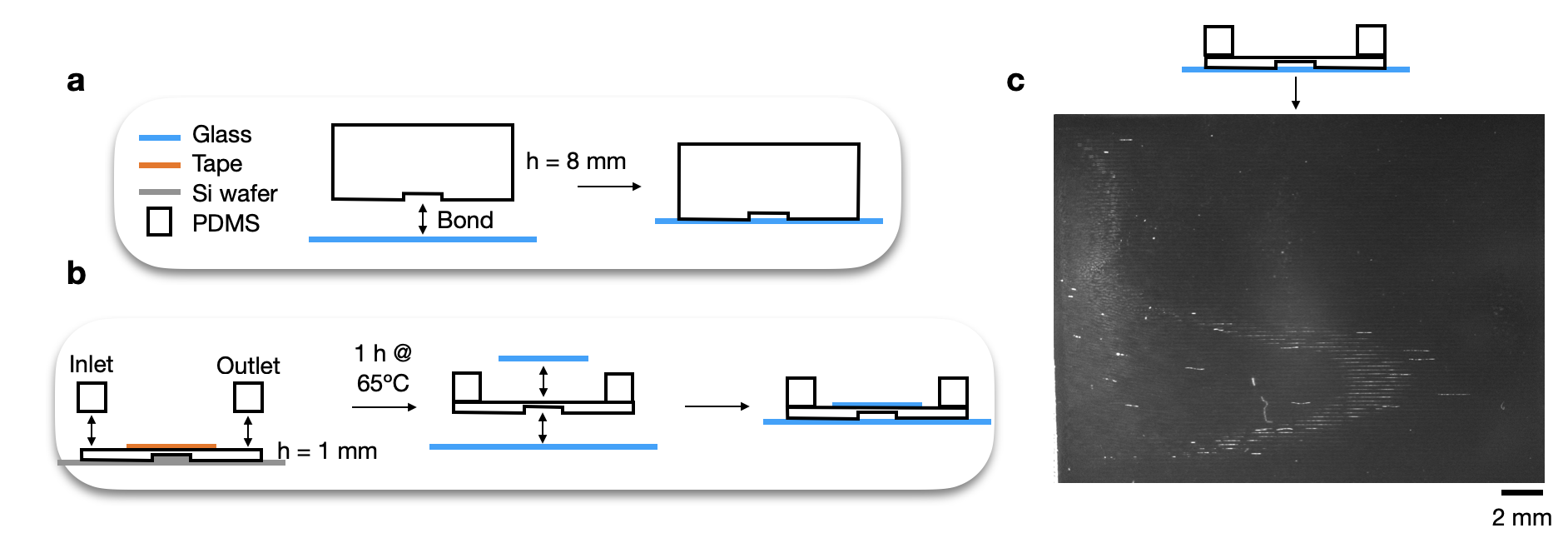


**SI Figure 5**. Fabrication steps for the unoptimized (**a**) and sandwiched thin (**b**) PDMS detection device. **a)** The 8 mm device is bonded to a glass slide after being cut out from the Si wafer and punched. **b)** To fabricate the thin device, we first bond 10x10x5 mm^3^ (lxwxh) PDMS posts to the back side of the device, while the PDMS is still on the Si wafer, aligning it with the inlet and outlet. We protect the detection region from plasma treatment with Kapton tape. After baking at 65 ºC for 1 hour, we cut out the device from the wafer, punch the inlet and outlets using a 1.5 mm biopsy punch, and tape it with Kapton tape to prevent the channels from deforming due to the flexibility of PDMS at such a low thickness. Next, we bond both sides of the PDMS device to glass, sandwiching the PDMS detection region. **c)** If we only bond one side of the thin PDMS to glass (i.e. the side with the features), the device delaminates at high (>300 ml/h) flow rates. The PDMS sandwich approach protects the device from delaminating.


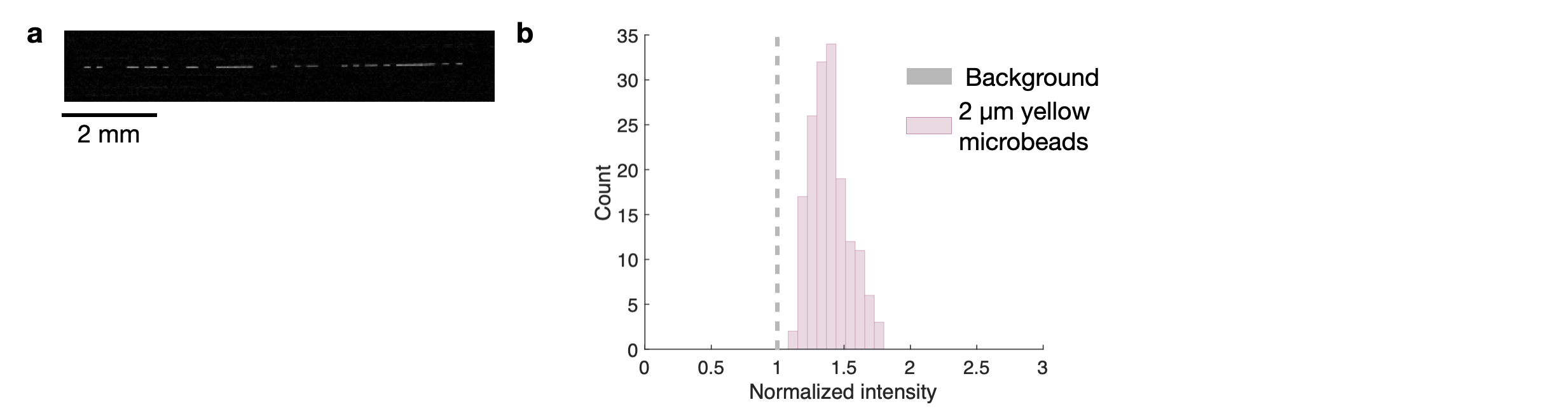


**SI Figure 6.** We interrogated d = 2 µm yellow microbeads (FCM-2052-2, Spherotech) with a single dye (ex/em = 470/490 nm) to assess our system’s compatibility with detecting smaller diameter beads while keeping the throughput constant. **a)** MLS-barcoded image of a bead. **b)** Average pixel values of individual bead streaks are above the background (N=162 beads).


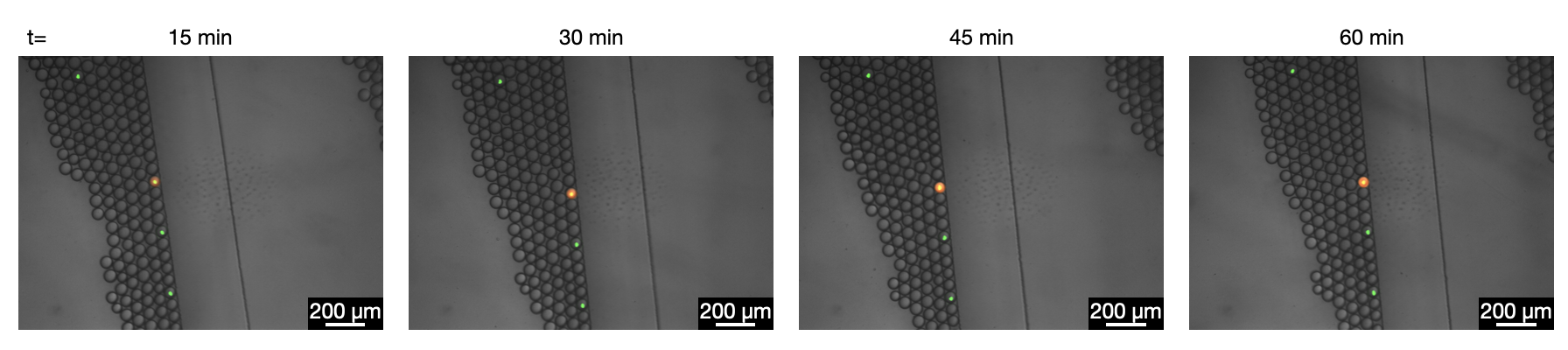


**SI Figure 7.** Time-lapse micrographs of droplets during the fluorescent substrate amplification step. The droplets contain biotin-antibody conjugated beads that have been incubated with 1:500,000 streptavidin-HRP, washed, and mixed with fluorescent substrate. Red fluorescent signal indicates the amplification of the fluorescent product as a result of the HRP-substrate reaction, which remains stable in the droplet while being in contact with neighboring droplets for one hour. t = incubation time point.


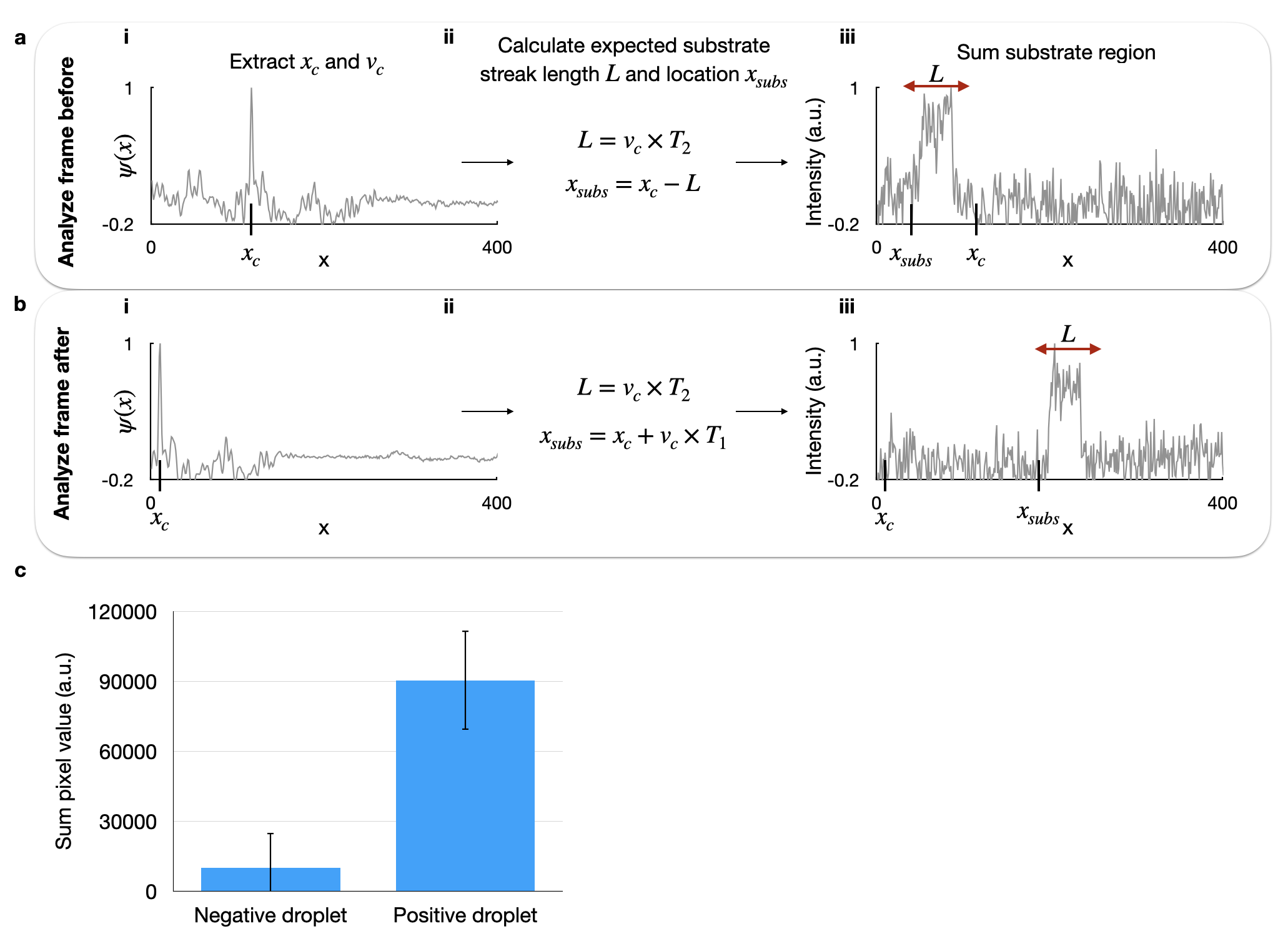


**SI Figure 8.** Schematic of automated fluorescent substrate detection. For each bead detected, we interrogate the frame before **(a)** and after **(b)** the bead frame to detect the substrate signal. To quantify substrate fluorescence, we **i)** first extract the location x_c_ and velocity v_c_ of the bead, **ii)** calculate the expected length L and location x_subs_ of the substrate streak based on the bead parameters, and **iii)** sum the pixel values along the calculated length of the substrate streak, starting at the expected location. **c)** Comparison of the average signal intensities of negative and positive droplets after thresholding, which is defined as 4 standard deviations above the background. N = 29572 negatives and 39 positives. Error bars represent standard deviation.


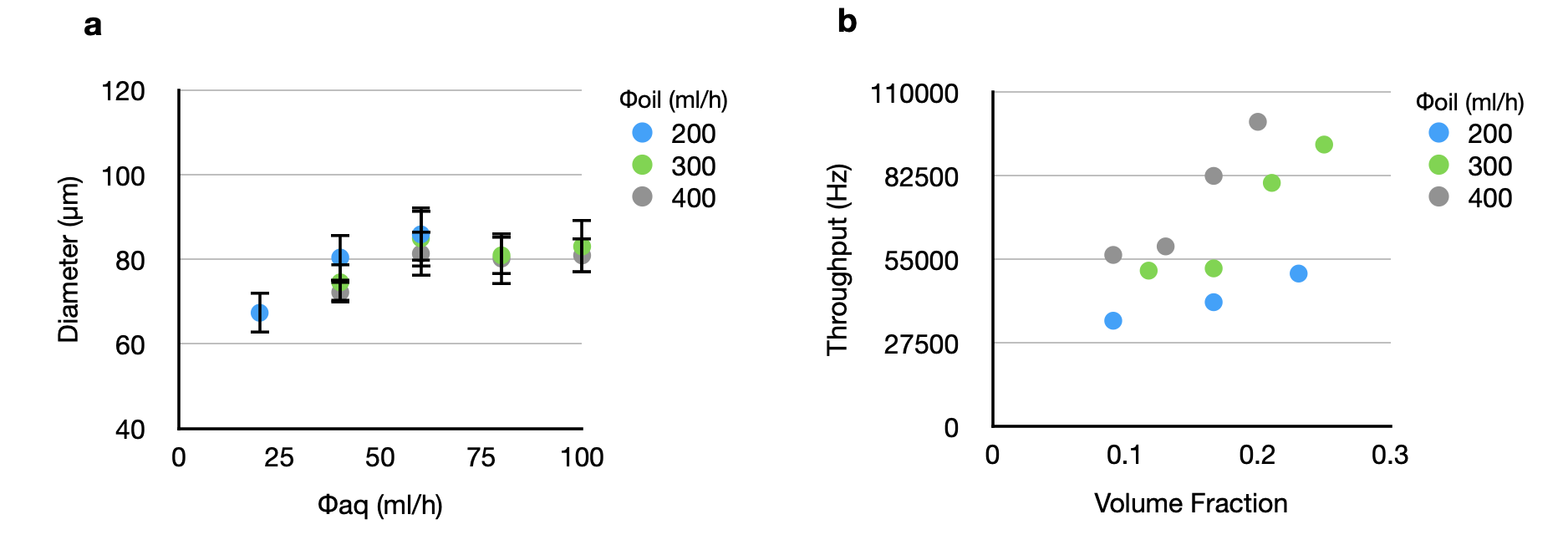


**SI Figure 9.** Millipede characterization. **a)** Droplet size is invariant to flow rate. **b)** Millipede device achieves a maximum throughput of 6x10^6^ droplets/minute.
